# Allelic variation in the indoleacetic acid-lysine synthase gene of the bacterial pathogen *Pseudomonas savastanoi* and its role in auxin production

**DOI:** 10.3389/fpls.2023.1176705

**Published:** 2023-06-06

**Authors:** Adrián Pintado, Hilario Domínguez-Cerván, Victoria Pastor, Marissa Vincent, Soon Goo Lee, Víctor Flors, Cayo Ramos

**Affiliations:** ^1^ Área de Genética, Facultad de Ciencias, Universidad de Málaga (UMA), Málaga, Spain; ^2^ Instituto de Hortofruticultura Subtropical y Mediterránea “La Mayora”, Consejo Superior de Investigaciones Científicas (IHSM-UMA-CSIC), Málaga, Spain; ^3^ Department of Biology, Biochemistry and Natural Sciences, Universitat Jaume I (UJI), Castelló de la Plana, Spain; ^4^ Department of Chemistry and Biochemistry, University of North Carolina Wilmington, Wilmington, NC, United States; ^5^ Department of Molecular and Cellular Biology, Kennesaw State University, Kennesaw, GA, United States

**Keywords:** allelic variation, auxin, IAA - Indole-3-acetic acid, IAA-lysine synthase, *Pseudomonas syringae*, *Pseudomonas savastanoi*

## Abstract

Indole-3-acetic acid (IAA) production is a pathogenicity/virulence factor in the *Pseudomonas syringae* complex, including *Pseudomonas savastanoi*. *P. savastanoi* pathovars (pvs.) genomes contain the *iaaL* gene, encoding an enzyme that catalyzes the biosynthesis of the less biologically active compound 3-indole-acetyl-ϵ-L–lysine (IAA–Lys). Previous studies have reported the identification of IAA–Lys in culture filtrates of *P. savastanoi* strains isolated from oleander (pv. nerii), but the conversion of IAA into a conjugate was not detectable in olive strains (pv. savastanoi). In this paper, we show the distribution of *iaaL* alleles in all available *P. savastanoi* genomes of strains isolated from woody hosts. Most strains encode two different paralogs, except for those isolated from broom (pv. retacarpa), which contain a single allele. In addition to the three previously reported *iaaL* alleles (*iaaL*_Psv_, *iaaL*_Psn_ and *iaaL*_Pto_), we identified *iaaL*_Psf_, an exclusive allele of strains isolated from ash (pv. fraxini). We also found that the production of IAA–Lys in *P. savastanoi* pv. savastanoi and pv. nerii depends on a functional *iaaL*_Psn_ allele, whereas in pv. fraxini depends on *iaaL*_Psf_. The production of IAA–Lys was detected in cultures of an olive strain heterologously expressing IaaL_Psn-1_, IaaL_Psf-1_ and IaaL_Psf-3_, but not when expressing IaaL_Psv-1_. In addition, *Arabidopsis* seedlings treated with the strains overproducing the conjugate, and thus reducing the free IAA content, alleviated the root elongation inhibitory effect of IAA. IAA–Lys synthase activity assays with purified allozymes confirmed the functionality and specificity of lysine as a substrate of IaaL_Psn-1_ and IaaL_Psf-3_, with IaaL_Psf-3_ showing the highest catalytic efficiency for both substrates. The IAA–Lys synthase activity of IaaL_Psn-1_ was abolished by the insertion of two additional tyrosine residues encoded in the inactive allozyme IaaL_Psv-1_. These results highlight the relevance of allelic variation in a phytohormone-related gene for the modulation of auxin production in a bacterial phytopathogen.

## 1 Introduction

Auxin balance is essential for the regulation of plant growth, development, and defense (Naseem et al., 2015). In plants, indole-3-acetic acid (IAA) is the most abundant natural auxin, and its homeostasis is regulated by a network of processes related to its biosynthesis, catabolism, signalling and transport. IAA is produced in plants through interlinked pathways sharing L-tryptophan (L-Trp) as a precursor. IAA levels can be regulated *via* conjugation to amino acids and sugars or *via* degradation, with only a small amount of free IAA remaining available (Normanly, 2010; Rosquete et al., 2012; Ljung, 2013). Various studies have identified the existence of IAA–amino acid conjugates in a wide variety of plants, such as IAA–aspartate (IAA–Asp), IAA–glutamate (IAA–Glu), IAA–alanine (IAA–Ala), IAA–glycine (IAA–Gly), IAA–valine (IAA–Val), IAA–leucine (IAA–Leu) and IAA–tryptophan (IAA–Trp). Only a few of these compounds, known as storage conjugates (IAA–Ala, IAA–Leu, IAA–Phe, and IAA–Val), are hydrolyzed back to free IAA by plant extracts or purified enzymes, modulating the concentration of free IAA and their distribution in plant organs (LeClere et al., 2002; Rampey et al., 2004; Ludwig-Müller, 2011). However, catabolism conjugates (IAA–Asp and IAA–Glu) are suggested to be involved in degradation pathways (Ludwig-Müller, 2011). In addition, IAA–Trp has been shown to be an inhibitor of IAA-dependent growth (Staswick et al., 2005).

Many plant-associated bacteria are also able to synthetize IAA using L-Trp as a precursor, modifying auxin balance and interfering with plant growth, organ development and defense responses. Thus, the production of IAA in phytopathogenic bacteria has been recognized as a pathogenicity or virulence factor (Spaepen and Vanderleyden, 2011; Patten et al., 2013). The indole-3-acetamide (IAM) pathway is the best-studied IAA biosynthetic pathway in bacteria. In this pathway, the enzymes tryptophan-2-monooxygenase and IAM hydrolase (which are encoded by the *iaaM* and *iaaH* genes, respectively) sequentially convert L-Trp to IAA (Magie et al., 1963; Kosuge et al., 1966). Other bacterial IAA pathways involve the intermediates indole-3-pyruvate, indole-3-acetonitrile, indole-3-acetaldehyde and tryptamine (Spaepen and Vanderleyden, 2011; Kunkel and Harper, 2017).

In bacteria, IAA can be metabolized to the less biologically active amino acid conjugate 3-indole-acetyl-ϵ-L–lysine (IAA–Lys) through the action of the IAA–Lys synthase enzyme, encoded by the *iaaL* gene (Hutzinger and Kosuge, 1968; Glass and Kosuge, 1986; Roberto et al., 1990). The natural production of IAA–Lys has only been shown in phytopathogenic bacteria of the *Pseudomonas syringae* species complex (Hutzinger and Kosuge, 1968; Evidente et al., 1986; Glass and Kosuge, 1986; Glass and Kosuge, 1988; Roberto et al., 1990; Castillo-Lizardo et al., 2015; Cerboneschi et al., 2016; Tegli et al., 2020). In fact, the codification of the *iaaL* gene is a common feature of most pathovars (pv.) of this bacterial complex (Glickmann et al., 1998; Xin et al., 2018). Although production of IAA–Lys has not been reported in plants, the bacterial *iaaL* gene constitutively expressed from a plant promoter can act as an anti-auxin gene in tobacco plants by reducing free-IAA levels (Romano et al., 1991).

The *P. syringae* complex, encompassing 15 different *Pseudomonas* species associated with plants and the water cycle, is divided into 13 phylogroups (Berge et al., 2014; Gomila et al., 2017). The species *Pseudomonas savastanoi* belongs to phylogroup 3, the only one including bacteria that cause tumorous overgrowths (knots) in woody hosts (Caballo-Ponce et al., 2017). The number of *iaaL* paralogs and their locations in the chromosome and/or native plasmids of *P. savastanoi* vary among pathovars and strains. The knot-forming *P. savastanoi* pathovars are *P. savastanoi* pv. mandevillae (Psm), *P. savastanoi* pv. nerii (Psn), *P. savastanoi* pv. retacarpa (Psr) and *P. savastanoi* pv. savastanoi (Psv), which include isolates from dipladenia (*Mandevilla* spp.), oleander (*Nerium oleander*), broom (*Retama sphaerocarpa*) and olive (*Olea europaea*), respectively (Gardan et al., 1992; Bull et al., 2010; Caballo-Ponce et al., 2017; Caballo-Ponce et al., 2021). Psv strains generally encode two chromosomal *iaaL* paralogs, whereas Psn strains usually contain the *iaaL* gene in plasmids (Comai et al., 1982; Glass and Kosuge, 1986; Caponero et al., 1995; Matas et al., 2009). Comparative nucleotide sequence analysis and restriction fragment length polymorphism (RFLP) showed that Psv strains encode two *iaaL* paralogs, *iaaL*_Psn_, identical to that encoded in plasmid pIAA1 from Psn strain EW 2009 (Roberto et al., 1990) and, *iaaL*_Psv_, exhibiting 93% identity to the ortholog gene (*iaaL*_Pto_) from *P. syingae* pv. tomato (Pto) (Matas et al., 2009). The *iaaL* gene is also found in the genomes of the Psm, Psr, and *P. savastanoi* pv. fraxini (Psf) strains (Moreno-Pérez et al., 2020; Caballo-Ponce et al., 2021), with the latter causing cankers accompanied by excrescences in ash (*Fraxinus excelsior*) (Janse, 1981; Gardan et al., 1992; Caballo-Ponce et al., 2017). However, the *iaaL* allelic variants encoded in Psf, Psm, Psr and most Psn strains have not yet been reported.

Production of IAA–Lys has only been detected in culture filtrates of Psn but not of Psv strains (Evidente et al., 1986; Glass and Kosuge, 1986). In agreement with these results, natural variations of the *iaaL*_Psv_ and *iaaL*_Psn_ sequences encoded by certain Psv strains have been reported and suggested to inactivate IAA–Lys synthase function (Matas et al., 2009; Rodríguez-Palenzuela et al., 2010). However, the functionality of the IAA–Lys synthases encoded by these *iaaL* alleles has not yet been reported. In contrast, *iaaL*_Psn_ knockout mutants of the PB213 and *Psn23* Psn strains are unable to produce IAA–Lys and show increased IAA levels (Glass and Kosuge, 1988; Cerboneschi et al., 2016; Tegli et al., 2020), demonstrating that IAA–Lys production in these two strains is dependent on their specific *iaaL* allelic variants. However, the impact of the *iaaL* gene and IAA–Lys production in virulence seems to be strain-dependent. While the *iaaL*_Psn_ mutant of Psn PB213 was found to cause virulence attenuation in oleander (Glass and Kosuge, 1988), the *Psn23 iaaL*_Psn_ mutant showed a hypervirulent phenotype (Glass and Kosuge, 1988; Cerboneschi et al., 2016). On the other hand, the replacement of *iaaL*_Pto_ by a kanamycin resistance cassette in Pto DC3000 was shown to cause virulence reductions in tomato plants (Castillo-Lizardo et al., 2015). The functionality and role in virulence of other *iaaL* allelic variants have not been reported so far.

Here, we show the distribution and number of *iaaL* alleles encoded in all publicly available genome sequences of *P. savastanoi* strains isolated from woody hosts. A comparison of *iaaL* nucleotide sequences and phylogenetic analyses of their encoded proteins revealed the existence of a fourth allele, *iaaL*_Psf_, exclusive to Psf strains. IAA and IAA–Lys production was tested in a selection of *P. savastanoi* strains belonging to all pathovars and encoding diverse combinations of *iaaL* alleles. The functionality of all four *iaaL* alleles from *P. savastanoi* strains and Pto DC3000, *iaaL*_Pto_, was assessed by heterologous expression in an olive strain not producing IAA–Lys, as well as with IAA–Lys synthase activity assays with purified IaaL allozymes. Finally, we demonstrate the inactivation of IaaL_Psn_ from a Psn strain by insertion of additional amino acid residues encoded in an inactive IaaL_Psv_ allozyme.

## 2 Experimental procedures

### 2.1 Phylogenetic analysis

Amino acid sequences of the IaaL proteins encoded in all 22 publicly available *P. savastanoi* genomes were identified with blastp and downloaded from the National Centre for Biotechnology Information (NCBI). Phylogenetic relationships were predicted using MEGAX (Kumar et al., 2018) with the maximum likelihood method and 100 bootstrap replicates to evaluate the tree topology. The sequence of IaaL_Pto_ from *P. syringae* pv. tomato DC3000 was used as an external group.

### 2.2 Bacterial strains and growth conditions

Bacterial strains were grown at 28°C (*P. savastanoi* strains) or 37 °C (*Escherichia coli* strains) in a lysogenic culture medium (LB) (Bertani, 1951) or a super optimal culture medium (SOB) (Hanahan, 1983). In addition, *P. savastanoi* strains were grown in a minimal mannitol–glutamate (MG) medium supplemented with ferric citrate (10 g/L of mannitol, 2 g/L of L-glutamic acid, 0.5 g/L of KH_2_PO_4_, 0.2 g/L of NaCl, and 0.2 g/L of MgSO_4_, pH 7) (Keane et al., 1970). The bacterial strains used in this study are indicated in **Table S1**. When necessary, culture media were supplemented with 10 µg/mL and 50 µg/mL of kanamycin (Km) for the *E. coli* and *Pseudomonas* strains, respectively.

### 2.3 Quantification of IAA and IAA–Lys and other conjugates

The production of IAA and IAA–Lys by *P. savastanoi* strains was quantified in exponentially growing MG medium cultures (optical density at 600 nm (OD_600_) of 0.5). Quantification was performed using an Acquity ultra-performance liquid chromatography system (UPLC; Waters, Mildford, MA, USA) coupled to a triple quadrupole mass spectrometer detector (TQD; Waters, Manchester, UK). Chromatographic separation was performed using a Kinetex C18 analytical column, with a particle size of 1.7 µm, 50 mm × 2.1 mm (Phenomenex, California, United States). A MeOH gradient in water with 0.01% HCOOH and a flow of 0.3 mL/min was applied for sample elution, following the parameters described in (Gamir et al., 2014). We mixed 1 mL of culture supernatants with MeOH/H_2_O (10:90 v/v), and indole acetic acid-d5 (IAA-d5) (WuXi LabNetwork, Massachusetts, USA) was used as internal standard to a final concentration of 100 ng/mL. IAA–Lys, used as a standard, was synthesized by WuXi App Tec, co., LTD (Wuhan, China). The concentration of IAA and IAA–Lys is expressed as μg per g of dry weight of the bacterial culture.

### 2.4 Construction of bacterial strains and plasmids

The plasmids and oligonucleotides used in this work are indicated in **Tables S2**, **S3**, respectively. For the heterologous expression of *iaaL* alleles in Psv NCPPB 3335, the open reading frames (ORF) of the different alleles were PCR-amplified using genomic DNA from their corresponding strains as templates, Q5 high-fidelity DNA polymerase (New England Biolabs, Hitchin, UK), and oligonucleotides *iaaL*–RBS-F and *iaaL*–RBS-R (**Table S3**). The resulting products were cloned into the constitutive expression vector pAMEX using the restriction enzyme sites included in the oligonucleotides (**Table S3**). The resulting plasmids were transferred to Psv NCPPB 3335 *via* electroporation, and transformants were selected in an LB–Km medium (Pérez-Martínez et al., 2007).

His-tagged constructs of IaaL allozymes were generated *via* the PCR amplification of wild-type *iaaL* templates using Q5 high-fidelity DNA polymerase and appropriate oligonucleotides (**Table S3**). The resulting fragments were subcloned into plasmid pET28a (**Table S2**) for C-terminal His-tagging. For the expression of His6-tagged IaaL proteins, each pET28a-IaaL construct was transformed into *E. coli* BL21 (**Table S1**).

The site-directed mutagenesis of *iaaL*_Psn-1_ on the pET28a::*iaaL*_Psn-1_ construct was generated using oligonucleotides Mut_YY_F and Mut_YY_R (**Table S3**) and the QuickChange II Site-Directed Mutagenesis Kit (Stratagene, USA) following the supplier’s instructions. The resulting vector, pET28a::*iaaL*_PsnYY_ (**Table S2**), encodes a His6-tagged IaaL_Psn-1_ protein containing an insertion of two tyrosine residues (Y_81_Y_82_) at the same positions as IaaL_Psv-1_.

### 2.5 *Arabidopsis* root elongation test

*Arabidopsis thaliana* Col-0 seeds were surface-sterilized with 1% bleach and rinsed three times in sterile distilled water. Subsequently, the seeds were stratified on Murashige–Skoog (MS) plates (Murashige and Skoog, 1962) with 10 g/L of agar (pH 5.7-5.8) and incubated at 4°C for 2 days to obtain uniform germination. Bacterial suspensions were obtained from LB overnight cultures, washed three times with 10 mM of MgSO_4_ and adjusted to an OD_600_ of 0.5, corresponding to approximately 10^8^ CFUs/mL. Then, *A. thaliana* seedlings were exposed to 20 µL of the bacterial suspensions located at 4 cm from the root tips (Qin et al., 2005; Cerboneschi et al., 2016). The plates were grown upright in a growth chamber at 22°C with a short-day photoperiod of 8 h of light and 16 h of darkness. Photographs were obtained after an additional 15 days of vertical growth, and root length and area was measured using ImageJ software. The mean root length and root area ± standard deviation was calculated for 10 to 15 seedlings.

### 2.6 Protein expression and purification

Transformed *E. coli* BL21 cells with the different pET28a plasmids expressing IaaL allozymes were incubated in LB medium overnight at 37 °C. Subsequently, a 1/50 dilution of this culture was made in fresh LB medium and incubated at 37 °C to an OD_600_ of 0.5. To optimize the production of the IaaL protein, different induction conditions were tested, and incubation for 4 h at 28 °C in the presence of 1 mM of isopropyl-β-D-1-thiogalactopyranoside (IPTG) was selected after verification *via* Western blot analysis (data not shown). After induction, *E. coli* BL21 cells were harvested by centrifugation, resuspended in lysis buffer (50 mM Tris, pH 8.0, 500 mM NaCl, 25 mM imidazole, 10% glycerol, and 1% Tween-20) and lysed by sonication (100W, 30kHz, 80% amplitude, three times for 1 min). The supernatant with the soluble protein obtained after 45 min of centrifugation at 16000g at 4°C was filtered through a Ni^2+^ chromatography column. The column was washed using washing buffer (lysis buffer without Tween-20) and subsequently eluted in elution buffer (washing buffer with 250 mM of imidazole). The eluted proteins were dialyzed using a dialysis membrane and a solution composed of 25 mM HEPES, 100 mM NaCl and 10% glycerol at pH 7.5. The concentration of extracted proteins were quantified using Bradford’s reagent and bovine serum albumin as a standard (Bradford, 1976).

### 2.7 Enzymes assays to determine kinetic parameters

The enzymatic activity of each IaaL allozyme (i.e., IaaL_Psn-1_, IaaL_Psf-3_, IaaL_Psv-1_, IaaL_Pto_ and IaaL_PsnYY_) was spectrophotometrically measured with an enzyme-coupled continuous spectrophotometric assay (i.e., the myokinase, pyruvate kinase, and lactate dehydrogenase system) by detecting the formation of AMP (Chen et al., 2010). The spectrophotometric signal, which was NADH reduction (ϵ_340 nm_ = 6220 M^−1^cm^−1^), was monitored at A_340nm_ using an EPOCH2 microplate spectrophotometer (BioTek, Vermont, USA). The standard assay conditions for IaaL allozymes were 50 mM Tris**·**HCl (pH 8.0), 100 mM KCl, 3 mM MgCl_2_, 1 mM ATP, 1 mM phosphoenolpyruvate, 0.2 mM NADH, 1 mM IAA, 5 mM lysine, 2 units of rabbit muscle myokinase, 4 units of rabbit muscle pyruvate kinase, and 4 units of rabbit muscle lactate dehydrogenase in a total volume of 200 μL at 25 °C. For the determination of steady-state kinetic parameters, reactions were performed in standard assay conditions with either fixed IAA (1.0 mM) and varied lysine (0.01–10 mM) or with fixed lysine (5.0 mM) and varied IAA (0.05–10 mM). The rate of IAA–Lys (product) formation was calculated based on the product/NADH conversion rate at which 1 molecule of IAA–Lys per 2 molecules of NADH formed. All resulting data were fit to the Michaelis–Menten equation, *v* = (*k*_cat_[S])/(*K*_m_ + [S]), using Prism (GraphPad).

## 3 Results

### 3.1 Distribution of IaaL allozymes in *P. savastanoi*

Bioinformatic analyses were performed to identify the IaaL variants encoded in the genomes of strains from all pathovars of *P. savastanoi*. blastp searches with the sequence of IaaL_Psn_ from *Psn23* against all 22 P*. savastanoi* genomes available at the NCBI identified 39 IaaL homologs, 34 of which shared 100% coverage and >90% identity with the query. In addition, five partial sequences were also found. Psv strain 0485, whose draft genome includes two incomplete *iaaL* sequences, was not included in these analyses (**Table 1**). While most Psm, Psn, Psv, and Psf strains encode two different IaaL allozymes, Psr strains encode a single IaaL protein. In silico analyses of the nucleotide sequences encoding all these proteins revealed that Psr strains encode an *iaaL*_Psn_ allele, here named *iaaL*_Psn-1_, identical to that also found in all four Psn strains, Psm Ph3, and seven of the nine Psv genomes. In addition, all Psn and Psv strains (except for Psn ICMP 13781), as well as Psm Ph3, encode an *iaaL*_Psv_ allele. Four out of the five analyzed Psf genomes encode two different *iaaL* alleles. However, in silico and electrophoresis analyses of an internal fragment of the *iaaL* gene revealed that these alleles cannot be differentiated from *iaaL*_Psn_ and *iaaL*_Psv_ using the PCR-RFLP procedure described by Matas et al. (2009) (**Figure S1**).

**Table 1:** Distribution of the *iaaL* genes in the *P. savastanoi* strains analyzed in this work.

Pathovar	Strain^a^	*iaaL* allele^b^	Protein ID	(TAC)n^c^	Reference
**pv. savastanoi**	**NCPPB 3335**	*iaaL*_Psv-1_	WP_002555797.1	6	(Moreno-Pérez et al., 2020)
		*iaaL*_Psn_ *_-_*_2_	WP_082301237.1		
	**DAPP-PG722**	*iaaL*_Psv_ *_-_*_2_	WP_031595740.1	4	(Moretti et al., 2014)
		*iaaL*_Psn_ *_-_*_1_	WP_031598440.1		
	PseNe107	*iaaL*_Psv_ *_-_*_2_	WP_031595740.1	4	(Bartoli et al., 2015)
		*iaaL*_Psn_ *_-_*_1_	WP_031598440.1		
	ICMP 4352	*iaaL*_Psv_ *_-_*_5_	WP_054082606.1	4	(Thakur et al., 2016)
		*iaaL*_Psn_ *_-_*_1_	WP_031598440.1		
	ICMP 13519	P	WP_019331605.1	4	(Dillon et al., 2019b)
		*iaaL*_Psn_ *_-_*_1_	WP_031598440.1		
	ICMP 1411	*iaaL*_Psv_ *_-_*_1_	WP_002555797.1	6	(Dillon et al., 2019b)
		*iaaL*_Psn_ *_-_*_2_	WP_082301237.1		
	0485_9	P	WP_197094824.1	4	(Dillon et al., 2019b)
		P	WP_020342755.1		
	ICMP 13786	*iaaL*_Psv-4_	WP_122260465.1	4	(Dillon et al., 2019b)
		*iaaL*_Psn-1_	WP_031598440.1		
	PVFi	*iaaL*_Psv_ *_-_*_2_	WP_031595740.1	4	(Turco et al., 2022)
		*iaaL*_Psn-1_	WP_031598440.1		
**pv. fraxini**	**CFBP 5062**	*iaaL*_Psv_ *_-_*_2_	WP_031595740.1	4	(Nowell et al., 2016)
		*iaaL*_Psf-1_	WP_060410024.1		
	**NCPPB 1006**	*iaaL*_Psf-3_	WP_095077645.1	4	(Moreno-Pérez et al., 2020)
	ICMP 9132	*iaaL*_Psv-6_	WP_122253379.1	4	(Dillon et al., 2019b)
		*iaaL*_Psf-1_	WP_060410024.1		
	ICMP 7712	P	WP_019331605.1	4	(Dillon et al., 2019b)
		*iaaL*_Psf-1_	WP_060410024.1		
	ICMP 9129	P	WP_019331605.1	4	(Dillon et al., 2019b)
		*iaaL*_Psf-2_	WP_122272660.1		
**pv. nerii**	**CFBP 5067**	*iaaL*_Psv-3_	WP_057442603.1	4	(Nowell et al., 2016)
		*iaaL*_Psn-1_	WP_031598440.1		
	***Psn23***	*iaaL*_Psv_ *_-_*_2_	WP_031595740.1	4	(Moreno-Pérez et al., 2020)
		*iaaL*_Psn-1_	WP_031598440.1		
	ICMP 16944	*iaaL*_Psv-2_	WP_019331605.1	4	(Dillon et al., 2019b)
		*iaaL*_Psn-1_	WP_031598440.1		
	ICMP 13781	*iaaL*_Psn-1_	WP_031598440.1	4	(Dillon et al., 2019b)
**pv. retacarpa**	CECT 4861	*iaaL*_Psn-1_	WP_031598440.1	4	(Moreno-Pérez et al., 2020)
	ICMP 16947	*iaaL*_Psn-1_	WP_031598440.1	4	(Dillon et al., 2019b)
	ICMP 16946	*iaaL*_Psn-1_	WP_031598440.1	4	(Dillon et al., 2019b)
**pv. mandevillae**	Ph3	*iaaL*_Psv-2_	WP_031595740.1	4	(Caballo-Ponce et al., 2021)
		*iaaL*_Psn-1_	WP_031598440.1		

a Bold names indicate *P. savastanoi* strains analyzed in terms of their production of IAA and IAA–Lys; Psv ICMP 4352 is synonym of NCPPB 639; Psm Ph3 syn. Psm CFBP 8832 (Caballo-Ponce et al., 2021).

b P, partial sequence.

c Number of L-tyrosine (Y) repeats encoded in the amino acid sequences of IaaL proteins.

**Figure 1:**
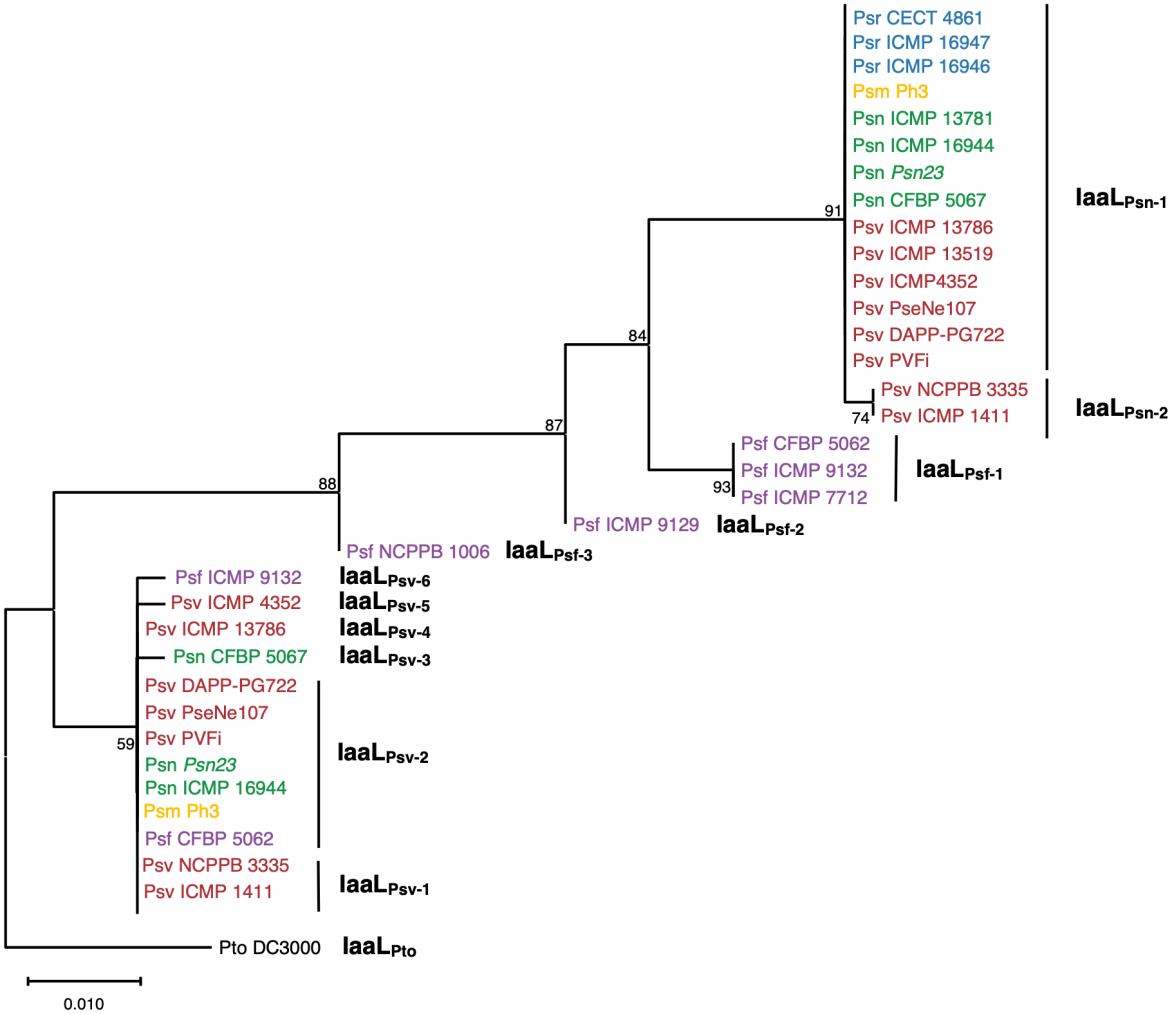
Phylogenetic distribution of IaaL allozymes encoded in the genomes of *P. savastanoi* strains isolated from woody hosts. The maximum likelihood tree was rooted with IaaL_Pto_ from *P. syringae* pv. tomato (Pto) DC3000. Values in nodes are bootstrap percentages from 100 replicates; the scale represents substitutions per site. Psv (red), Psn (green), Psf (purple), Psr (blue) and Psm (yellow) refer to the *P. savastanoi* pathovars savastanoi, nerii, fraxini, retacarpa and mandevillae, respectively.

To analyze the phylogenetic relationships of IaaL allozymes encoded in the *P. savastanoi* strains, all 34 complete sequences were used (**Table 1**), and the phylogenetic tree was rooted using the sequence of IaaL_Pto_ from Pto DC3000. The tree showed five well-differentiated clades, two of which corresponded to IaaL_Psn_ and IaaL_Psv_ and included all allozymes encoded by Psn, Psv, Psr and Psm strains, as well as two IaaL_Psv_ proteins encoded by the CFBP 5062 and ICMP 9132 Psf strains. However, the remaining five IaaL sequences found in the Psf strains, here named IaaL_Psf_, clustered in a monophyletic branch together with the IaaL_Psn_ clade but separated into three additional clades (**Figure 1**).

A detailed analysis of the amino acid sequences of these 34 IaaL proteins identified a total of 12 different allozymes according to the codification of unique amino acid residues. Two IaaL_Psn_ variants encoding six exclusive residues were found: IaaL_Psn-1_, present in Psm Ph3 and most Psn, Psv and Psr strains, and IaaL_Psn-2_, which contains a 9-amino-acid N-terminal deletion and was only found in two olive isolates (Psv NCPPB 3335 and Psv ICMP 1411). Six IaaL_Psv_ variants sharing three exclusive residues and distributed among all pathovars, except for Psr, were also identified. In addition, IaaL_Psf_ sequences showed two unique residues (Lys_16_ and Met_51_) and were separated into three variants (IaaL_Psf-1_, IaaL_Psf-2_ and IaaL_Psf-3_) (**Table 1** and **Figure S2**), which corresponded with the three IaaL_Psf_ clades included in the phylogenetic tree (**Figure 1**).

In summary, *P. savastanoi* strains isolated from the same host share the same array of IaaL allozymes. While Psv, Psn and Psm strains encode two allozymes of the IAAL_Psn_ and IAAL_Psv_ groups, Psf strains harbor IAAL_Psv_ and are the only ones encoding an IAAL_Psf_ allozyme. In contrast, Psr strains encode a single IaaL_Psn_ variant.

### 3.2 Production of IAA and IAA–Lys by *P. savastanoi* strains

**Figure 2:**
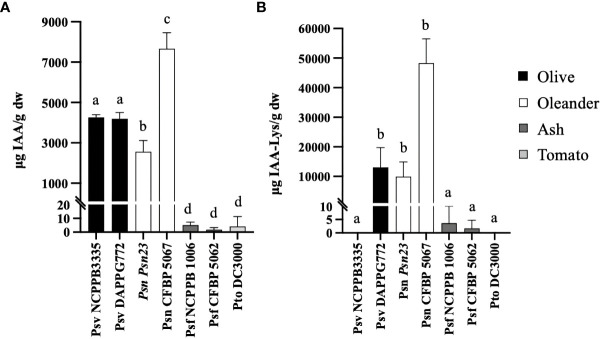
IAA **(A)** and IAA–lysine **(B)** concentrations in culture supernatants of representative *P. savastanoi* strains and *P. syringae* pv. tomato (Pto) DC3000 grown in MG medium. Each bar corresponds to the mean of three biological replicates, and the error bars represent the standard deviation. Different letters indicate means that are significantly different using ANOVA test followed by the Bonferroni t-test (p < 0.05).

The ability to produce IAA–Lys by *P. savastanoi* strains has been only reported for Psn strains (Evidente et al., 1986). Here, we analyzed the production of IAA and IAA–Lys in a selection of *P. savastanoi* strains encoding different combinations of *iaaL* alleles. The selected strains (**Figure 2** and **Table 1**) were found to encode three diverse *iaaL*_Psv_ alleles (*iaaL*_Psv-1_, *iaaL*_Psv-2_ and *iaaL*_Psv-3_), two *iaaL*_Psn_ alleles (*iaaL*_Psn-1_ and *iaaL*_Psn-2_) and two different *iaaL*_Psf_ alleles (*iaaL*_Psf-1_ and *iaaL*_Psf-3_). Pto DC3000 (*iaaL*_Pto_ allele) was also included in these analyses.

In agreement with previous reports (Smidt and Kosuge, 1978; Comai and Kosuge, 1980; Surico et al., 1985; Glickmann et al., 1998; Aragón et al., 2014), Psv and Psn strains grown in MG medium produced high amounts of IAA, reaching concentrations in the supernatant ranging from approximately 4193 to 7668 µg IAA/g of dry weight (dw). However, the concentration of IAA in spent supernatants of Pto DC3000 and Psf strains, which lack the *iaaM* and *iaaH* genes (Glickmann et al., 1998; Iacobellis et al., 1998; Moreno-Pérez et al., 2020), was about three orders of magnitude lower, from approximately 4.13 to 1.64 µg IAA/g dw (**Figure 2A**). In addition, except for Psv NCPPB 3335 (*iaaL*_Psn-2_ and *iaaL*_Psv-1_) and Pto DC3000 (*iaaL*_Pto_), all other strains produced detectable amounts of IAA–Lys under the conditions tested. The production of IAA–Lys was positively correlated with the amount of IAA produced by the Psn and Psf strains, as well as by strain Psv DAPPG772 (*iaaL*_Psn-1_ and *iaaL*_Psv-2_). However, the concentrations of IAA–Lys found in the supernatant of the Psf strains was approximately 1,000 times lower than that of the Psn strains (**Figure 2B**). Thus, the IaaL allozymes produced by *iaaL*_Psn-2_, *iaaL*_Psv-1_ and *iaaL*_Pto_ appear to be functionally compromised under the conditions tested. This is likely due to their sequence variations with respect to the fully functional enzymes (**Figure 2**), including a 9-amino-acid N-terminal deletion in *iaaL*_Psn-2_ and an insertion of two tyrosine residues at position 81 in *iaaL*_Psv-1_. These results, together with the functional inactivation of *iaaL*_Psv-2_ in Psn23 suggested by Cerboneschi et al. (2016), indicate that the production of IAA–Lys in the strains tested is mainly dependent on the codification of an *iaaL*_Psn-1_ allele in Psv DAPP-PG722 and Psn Psn23 (*iaaL*_Psn-1_, *iaaL*_Psv-2_), an *iaaL*_Psf-1_ allele in Psf CFBP 5062 (*iaaL*_Psv-2_, *iaaL*_Psf-1_) or an *iaaL*_Psf-3_ allele in Psf NCPPB 1006 (*iaaL*_Psf-3_). In addition, the IAA–Lys production in Psn CFBP 5067 (*iaaL*_Psn-1_ and *iaaL*_Psv-3_) could be dependent on *iaaL*_Psn-1_ and *iaaL*_Psv-3_ or *iaaL*_Psn-1_ alone.

### 3.3 Functional evaluation of IaaL allozymes

**Figure 3:**
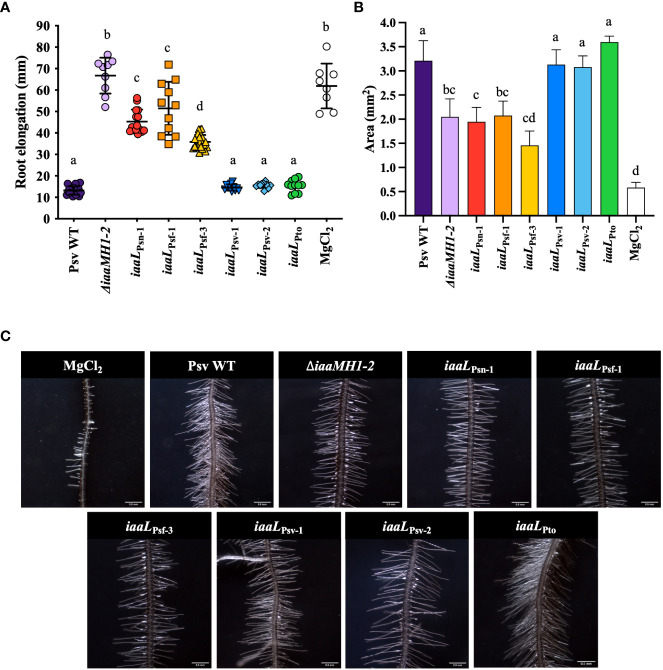
Root elongation **(A)**, root area **(B)**, and root hair formation **(C)** in *A. thaliana* Col-0 seedlings grown on vertical plates in the presence of Psv NCPPB 3335 strains overexpressing diverse *iaaL* alleles from the constitutive promoter *nptII.* The Psv NCPPB 3335 (Psv WT) and Psv *ΔiaaMH1-2* (*ΔiaaMH1-2*) strains were used as controls for the high and low production of free IAA, respectively. The bars represent the mean ± standard deviation for 9 to 15 seedlings. Different letters indicate means that are significantly different using ANOVA test followed by the Bonferroni t-test (p < 0.05).

Nine different *iaaL* alleles were selected for further functional analyses regarding their possible contribution to IAA–Lys production (*iaaL*_Psn-1_, *iaaL*_Psf-1_ and *iaaL*_Psf-3_) or their low or unknown ability to produce the conjugate (*iaaL*_Psv-1_, *iaaL*_Psv-2_, *iaaL*_Psv-3_, *iaaL*_Psv-4_, *iaaL*_Psv-5_ and *iaaL*_Pto_). All nine alleles were cloned in plasmid pAMEX under the control of the constitutive *nptII* promoter (**Table S2**) and were heterologously expressed in Psv NCPPB 3335, a strain that does not produce IAA–Lys (**Figure 2B**). The effect of the overexpression on the pool of IAA and IAA–Lys produced by these strains was first analyzed using an *Arabidopsis* root elongation assay based on the inhibitory effect of exogenous IAA on primary root and hypocotyl elongation (Qin et al., 2005). After 15 days, the mean length of the main root of the wild-type-treated (Psv NCPPB 3335) seedlings was 13.2 ± 1.92 cm, approximately 5-fold lower than that obtained for untreated seedlings, suggesting an inhibitory effect of the IAA produced by the strain on root development. In contrast, *Arabidopsis* seedlings treated with Psv Δ*iaaMH1-2*, a knockout mutant derived from Psv NCPPB 3335 producing concentrations of IAA 40 times lower than the wild-type strain (Aragón et al., 2014), showed root mean lengths similar to those of the untreated control (66.7 ± 8.4 cm). In addition, seedlings treated with the strains overexpressing *iaaL*_Psn-1_, *iaaL*_Psf-1_, and *iaaL*_Psf-3_ showed lower root mean lengths (45.34 ± 5.55 cm, 51.49 ± 12.33 cm, and 35.85 ± 3.24, respectively) than the wild-type control. However, the root mean lengths of seedlings exposed to Psv NCPPB 3335 overexpressing *iaaL*_Psv-1_, *iaaL*_Psv-2_, *iaaL*_Pto_ (**Figure 3A**), *iaaL*_Psv-4_, or *iaaL*_Psv-5_ (**Figure S3**) were similar to the wild-type control. These results suggest than *iaaL*_Pto_ and none of these *iaaL*_Psv_ alleles had a significant effect on the concentration of free IAA and IAA–Lys.

In addition to the observed inhibition of *Arabidopsis* root length following exposure to the IAA-producing *P. savastanoi* strains, the massive development of lateral roots was also observed in seedlings treated with either wild-type Psv NCPPB 3335 or the strains expressing *iaaL*_Psv-1_, *iaaL*_Psv-2_, or *iaaL*_Pto_. These results further support the inability or low ability of these three alleles to transform IAA into IAA–Lys. In fact, the mean root areas of seedlings treated with any of these strains were approximately 6 times that of the untreated control (0.58 ± 0.11 cm). Conversely, seedlings treated with strains accumulating lower concentrations of IAA than the wild-type control (Psv Δ*iaaMH1-2* and those overexpressing *iaaL*_Psn-1_, *iaaL*_Psf-1_ and *iaaL*_Psf-3_) showed smaller mean root areas (**Figures 3B, C**).

**Figure 4:**
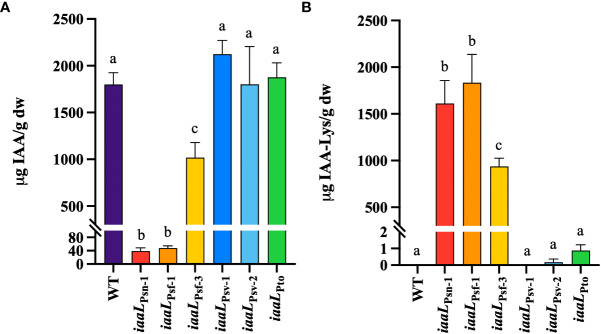
Quantification of IAA **(A)** and IAA–lysine **(B)** levels produced in culture supernatants of Psv NCPPB 3335 strains overexpressing different *iaaL* alleles under the control of the constitutive promoter *nptII*. Alleles were amplified from Psv NCPPB 3335 (*iaaL*_Psv-1_), Psn *Psn23* (*iaaL*_Psn-1_ *, iaaL*_Psv-2_), Psf CFBP 5062 (*iaaL*_Psf-1_), Psf NCPPB 1006 (*iaaL*_Psf-3_) and Pto DC3000 (*iaaL*_Pto_). WT, wild-type Psv NCPPB 3335. Bars represent the mean ± standard deviation of three biological replicates analyzed in duplicate. Different letters indicate means that are significantly different using ANOVA test followed by the Bonferroni t-test (p < 0.05).

To establish a correlation between root elongation and the IAA–Lys synthase activity of the diverse alleles expressed in Psv NCPPB 3335, the levels of IAA and IAA–Lys produced by a selection of these strains were quantified using UPLC–TQD. Overexpression of the three most active IaaL allozymes in strain NCPPB 3335 reduced the concentrations of free IAA in culture supernatants by approximately 1.8-fold (*iaaL*_Psf-3_) to 40-fold (*iaaL*_Psn-1_, *iaaL*_Psf-1_) (**Figure 4A**). This reduction was equivalent to the observed increase in the pool of IAA–Lys accumulated by the overexpressing derivative strains, further supporting the functionality of these three allozymes as IAA–Lys synthases (**Figure 4B**). Conversely, the overexpression of *iaaL*_Psv-1_, *iaaL*_Psv-2_, and *iaaL*_Pto_ did not have a significant effect on the concentration of the IAA or IAA–Lys produced by Psv NCPPB 3335 (**Figure 4**), further supporting the low activity or dysfunctionality of these allozymes. In summary, *iaaL*_Psn-1_, *iaaL*_Psf-1_ and *iaaL*_Psf-3_ encode functional IAA–Lys synthases that are able to synthesize IAA–Lys, thus withdrawing IAA from the medium. However, the allozymes encoded by *iaaL*_Psv-1_, *iaaL*_Psv-2_, and *iaaL*_Pto_, as well as *iaaL*_Psv-3_, *iaaL*_Psv-4_, and *iaaL*_Psv-5_, are likely inactive or have very low activity under the conditions tested.

### 3.4 Biochemical analysis of IaaL allozymes

**Figure 5:**
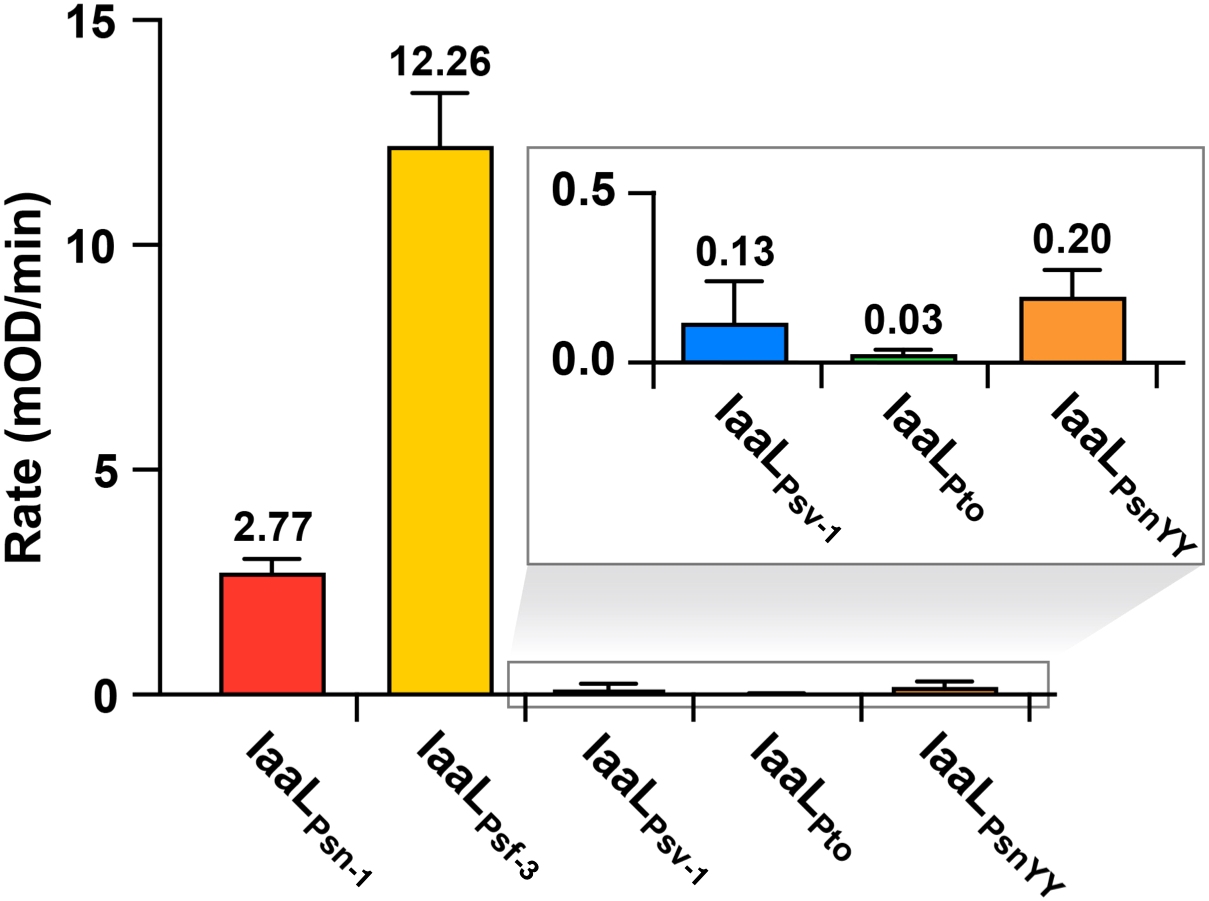
Specific activity of IaaL proteins encoded by the *iaaL*_Psn-1_, *iaaL*_Psf-3_, *iaaL*_Psv-1_, *iaaL*_Pto_ and *iaaL*_PsnYY_ alleles in the presence of 1 mM IAA and 5 mM lysine. Bars represent the mean ± standard deviation of three independent experiments.

IaaL allozymes are classified as members of the adenylating firefly luciferase (ANL) enzyme superfamily, containing a large N-terminal ANL domain with a smaller C-terminal domain (Sundlov et al., 2012). To further understand the molecular basis of IaaL enzymes, a biochemical analysis of four IaaL allozymes (IaaL_Psn-1_, IaaL_Psf-3_, IaaL_Psv-1_ and IaaL_Pto_), was performed. N-terminal hexahistidine-tagged IaaL allozymes were expressed in *E. coli* and purified with nickel-affinity and size-exclusion chromatography. Initial *in vitro* assays of purified IaaL_Psn-1_, IaaL_Psf-3_, IaaL_Psv-1_ and IaaL_Pto_ were performed with known substrates, IAA and lysine, to verify their IAA–amino synthetase activity, particularly IAA–Lys synthetase activity (**Figure 5**). IaaL_Psf-3_ showed the highest specific activity (12.26 mOD/min), which was 5-fold higher than IaaL_Psn-1_ (2.77 mOD/min) when IAA and lysine were provided as substrates. Compared with the other two IaaL enzymes, IaaL_Psv-1_ (0.13 mOD/min) and IaaL_Pto_ (0.03 mOD/min) showed 100- and 400-fold lower activity values, respectively (**Figure 5**). However, none of the IaaL enzymes showed detectable activity in the presence of 1 mM of IAA–Lys, indicating a clear preference for IAA–Lys formation without reverse reactions (data not shown). Additionally, the amino acid substrate specificities of three of these four allozymes (IaaL_Psn-1_, IaaL_Psf-3_ and IaaL_Psv-1_) were examined under standard enzyme assay conditions using all 20 amino acids, including lysine (**Figure S4**). Similar to the *in vivo* analysis (**Figure 4**), spectrophotometric assays of IaaL allozymes identified the highest enzymatic activity with lysine as a substrate while the other amino acids showed either significantly low or no detectable signal levels (**Figure S4**).

The steady-state kinetic analysis of IaaL with IAA and lysine confirmed differences in the enzymatic activity of the four IaaL allozymes. In the presence of variable concentrations of IAA, IaaL_Psf-3_ and IaaL_Psn-1_ followed the Michaelis–Menten kinetics, obtaining *V*_max_ of 53.3 ± 3.4 nmol/min mg and 10.6 ± 1.0 nmol/min mg, respectively. However, the kinetic parameters of IaaL_Psv-1_ and IaaL_Pto_ were not determined due to their low enzymatic activities. Although IaaL_Psn-1_ showed higher affinity for IAA than IaaL_Psf-3_ (*K*_m_ values of 617 ± 158 μM and 836 ± 142 μM, respectively), IaaL_Psf-3_ had the highest catalytic efficiencies (*V*_max_/*K*_m_), being 20- (for IAA) and 16-fold (for lysine) higher than those of IaaL_Psn-1_ (**Table 2**).

**Table 2:** Steady-state kinetic analysis of IaaL_Psn-1_ and IaaL_Psf-3_ with IAA and lysine.

Protein	Substrate	*V*_max_ (nmol/min mg)	*K*_m_ (μM)	*V*_max_/*K*_m_ (nmol/min mg mM)
IaaL_Psn-1_	IAA	10.6 ± 1.0	617 ± 158	17.2
	Lys	10.7 ± 1.3	1487 ± 431	7.2
IaaL_Psf-3_	IAA	53.3 ± 3.4	836 ± 142	363.7
	Lys	37.5 ± 1.7	320 ± 45	117.1

Assays were performed as described in the Experimental Procedures section. Average values ± S.D. (n = 3) are shown.

Overall, a series of *in vitro* biochemical analyses suggested that, of the four *iaaL* alleles containing typical ANL domains and predicted to function as IAA–amido synthetases, only IaaL_Psn-1_ and IaaL_Psf-3_ are capable of producing IAA–Lys at physiologically relevant concentrations under the conditions tested.

### 3.5 Inactivation of IaaL_Psn-1_ by insertion of two tandem tyrosine residues

Psv strains isolated in diverse geographical locations contain an *iaaL*_Psv_ allele exhibiting a variable number (3 to 15) of trinucleotide TAC tandem repeats that are in frame and located immediately after four TAC tyrosine codons (Y_77_ to Y_80_). This motif, which remains stable after bacterial propagation in olive plants, was suggested to be a mechanism for the inactivation of IAA–Lys synthetase activity in Psv (Matas et al., 2009). In agreement with these hypotheses, IaaL_Psv-1_, encoding two additional tyrosine residues (Y_81_ and Y_82_ (**Figure S2**)), showed a very low specific activity (**Figure 5**). To test this hypothesis, we performed site-directed mutagenesis of the active IAA–Lys synthase IaaL_Psn-1_ to introduce Y_81_ and Y_82_ into its amino acid sequence, generating IaaL_PsnYY_. The specific activity of IaaL_PsnYY_ (0.2 mOD/min), calculated as described above, was 0.07 times that of IaaL_Psn-1_ (**Figure 5**). Thus, the insertion of two additional tyrosine residues into IaaL_Psn-1_, at the same position as in IaaL_Psv-1_, caused the inactivation of the IAA–Lys synthase function of this allozyme.

## 4 Discussion

Functional studies of the allelic variants of virulence genes in the *P. syringae* complex include type III secretion system effectors (Lindeberg et al., 2006; Lindeberg et al., 2009; Baltrus et al., 2011; Lee et al., 2012; Lindeberg et al., 2012; Monteil et al., 2016; Dillon et al., 2019a; Moreno-Pérez et al., 2020), and the allelic diversity of two distinct *P. syringae* flagellin epitopes (Clarke et al., 2013). However, the allelic diversity of phytohormone-related genes has not been functionally analyzed in *P. syringae* or other bacterial phytopathogens.

### 4.1 *P. savastanoi* strains isolated from the same host share the same array of IaaL allozymes

In agreement with previous reports (Matas et al., 2009; Moreno-Pérez et al., 2020), we found that *P. savastanoi* strains isolated from woody hosts contain one or two *iaaL* alleles (**Figure 1** and **Table 1**), a gene exclusive to the *P. syringae* complex present in most of its pathovars regardless of whether they contain the *iaaMH* operon or not (Glickmann et al., 1998; Xin et al., 2018). In addition to the three previously described variants of this gene, *iaaL*_Psn_, *iaaL*_Psv_, and *iaaL*_Pto_ (Matas et al., 2009; Castillo-Lizardo et al., 2015; Moreno-Pérez et al., 2020), we showed here that Psf strains encode *iaaL*_Psf_, an allele restricted to this pathovar (**Table 1** and **Figure 1**). Psf strains, which are non-tumorigenic but induce cankers in ash, produce low concentrations of IAA (**Figure 2A**) using an unknown pathway independent of the *iaaMH* operon (IAM pathway). Conversely, strains of the tumorigenic *P. savastanoi* pathovars—Psn, Psm, Psv and Psr—produce high concentrations of IAA using the IAM pathway (Gardan et al., 1992; Glickmann et al., 1998; Moreno-Pérez et al., 2020) and encode a functional *iaaL*_Psn-1_ allele. Psv strains NCPPB 3335 and ICMP 1411 are an exception, as they contain *iaaL*_Psn-2_, likely encoding a low-activity or non-functional IAA–Lys synthase (**Figure 4**). Psv genomes are phylogenetically distributed into two different clades: Psv NCPPB 3335 and ICMP 1411 clustered with Psr whereas the remaining Psv strains analyzed here clustered with Psn (Moreno-Pérez et al., 2020). In addition, these two Psv strains are likely to be the only ones among all analyzed strains that are not able to produce IAA–Lys, as they also encode *iaaL*_Psv-1_ (**Table 1**), a low-activity or inactive IAA–Lys synthase (**Table 2**).

### 4.2 *P. savastanoi* strains from olive and ash can also produce IAA-Lys

Although previous studies reported that Psv strains do not produce IAA–Lys (Evidente et al., 1986; Glass and Kosuge, 1986), our results demonstrate that certain Psv strains, e.g. DAPP-PG722 (**Figure 2**), can synthesize this IAA conjugate. Therefore, our results suggest that other Psv strains encoding *iaaL*_Psn-1_ (**Table 1**) might also produce IAA–Lys. Nevertheless, strain ICMP 4352 (**Table 1**) was reported as not producing IAA–Lys (Evidente et al., 1986) although we identified an *iaaL*_Psn-1_ allele in its genome, suggesting the occurrence of other factors preventing synthesis of IAA-Lys. Thus, the Psv strains previously analyzed by other authors could all encode inactive IaaL allozymes or, for different reasons, produce concentrations of IAA–Lys that were undetectable in comparison with the amounts produced by Psn strains. In fact, a higher expression of *iaaL* is expected for Psn strains, since they carry this gene on plasmids, while all analyzed Psv strains encode *iaaL* in the chromosome (Glass and Kosuge, 1986).

Our data indicate that the genomic location of the *iaaL* gene might be correlated with its functionality. Thus, *iaaL* is encoded on the chromosome of strains Psv NCPPB 3335 and Pto DC3000 (Buell et al., 2003; Matas et al., 2009) and we did not detect production of IAA-Lys. The plasmid or chromosomal localization of *iaaL* has not been reported in the other five strains shown here to produce IAA-Lys. However, bioinformatic analyses of the draft genome sequences of these strains showed that their *iaaL*_Psv_ alleles are located in the proximity of chromosomally-encoded genes. In contrast, their functional *iaaL*_Psn-1_ (Psv DAPP-PG722, Psn CFBP 5067 and Psn Psn23), *iaaL*_Psf-1_ (Psf CBBP 5067) or *iaaL*_Psf-3_ (Psf NCPPB 1006) alleles were found near the *matE* gene and transposase-coding sequences (data not shown), a genomic context resembling that of the plasmid-encoded *iaaL* gene from Psn strain EW 2009 (Roberto et al., 1990). The product of *matE* (orf-1 identified by Roberto et al., 1990), is a multidrug and toxic compound extrusion (MATE) family transporter involved in the efflux of IAA and IAA–Lys in *Psn23*. Thus, it has been suggested that *matE*, in combination with *iaaL*, contributes to maintain IAA homeostasis by both regulating IAA efflux and generating the less biologically active compound IAA-Lys (Tegli et al., 2020).

### 4.3 Production of IAA-Lys depends on the alloenzymes IaaL_Psn-1_ or IaaL_Psf_ and the concentration of pathogen-produced IAA

The production of IAA–Lys was found to be correlated with the amounts of IAA produced by the Psv and Psn strains encoding IaaL_Psn-1_ and the Psf strains carrying IaaL_Psf-1_ or IaaL_Psf-3_, but not with Psv NCPPB 3335 (**Figure 2**). However, all derivatives of Psv NCPPB 3335 constitutively expressing IaaL_Psn-1_, IaaL_Psf-1_ or IaaL_Psf-3_ produced high concentrations of IAA–Lys (**Figure 4**). These results suggest that the low levels of IAA–Lys detected in Psf might have been due to the small concentration of the IAA produced by the strains rather than to the low activity of the allozymes IaaL_Psf-1_ and IaaL_Psf-3_. In fact, these three allozymes alleviated the inhibitory effect of IAA on the elongation of *Arabidopsis* roots by two to three times (**Figure 3**). Moreover, IaaL_Psf-3_ showed a catalytic efficiency for IAA about 20 times higher than that of IaaL_Psn-1_ (**Table 2**).

According to our results, IaaL_Psv-1_, IaaL_Psv-2_ and IaaL_Pto_ are likely inactive or have very low activity under the conditions tested, since i) the strains carrying these allozymes did not produce IAA–Lys (**Figure 1** and **Table 1**), ii) their heterologous expression in Psv NCPPB 3335 cultures did not result in an increased production of IAA or IAA–Lys (**Figure 3**) or in the elongation of *Arabidopsis* roots (**Figure 4**), and iii) purified IaaL_Psv-1_ and IaaL_Pto_ were not able to produce IAA–Lys at physiologically relevant concentrations (**Figure 5** and not tested for IaaL_Psv-2_). Although strains Psf and Pto DC3300 produced similar IAA amounts under the conditions tested (**Figure 2**), the purified IaaL_Psf-3_ allozyme had a higher enzymatic activity than IaaL_Pto_, which is likely due to the variability in their amino acid sequences (**Figure S2**). Nevertheless, considering that tryptophan is the precursor of IAA biosynthesis in both *P. savastanoi* and Pto DC3000, it could be possible that exogenous addition of tryptophan to the culture medium would result in the production of detectable amounts of IAA–Lys by the tested strains.

### 4.4 Inactivation of IaaL_Psn_ from a Psn strain by site-directed mutagenesis

Allozymes of the IaaL_Psv_ group had a similar enzymatic activity to those of the IaaL_Pto_ allozyme, including the nonsignificant IAA–Lys synthase activity shown by purified IaaL_Psv-1_ (**Figure 5**). These results were expected, considering the inactivation of IaaL_Psn-1_ after the insertion of the two IaaL_Psv-1_-specific tyrosine residues (**Figure 5**). Expansion and contraction of the number of short tandem repeat sequences in protein-coding regions due to slipped-strand mispairing during DNA synthesis has been related to bacterial adjustment to environmental changes (Bichara et al., 2006), and was reported to also occur in bacterial phytopathogens (Jock et al., 2003). Other IaaL_Psv_ allozymes analyzed in this study, e.g. IaaL_Psv-2_, IaaL_Psv-3_, IaaL_Psv-4_, and IaaL_Psv-5_, are also non-functional (**Figures 4**, **S3**) despite conserving the canonical four tyrosine motif, suggesting that additional amino acid changes must be responsible for their inactivity (**Figure S2**). All these enzymes, encoded in the strains of all *P. savastanoi* pathovars except for Psr, form a monophyletic branch in the IaaL phylogeny that is well-separated from all other allozymes (**Figure 1**). Thus, genes coding for inactive or low-activity IaaL_Psv_ allozymes might have been transmitted *via* horizontal transfer. In fact, although the *iaaL* gene is ancestral to the *P. syringae* complex (Ramos et al., 2012), the IaaL phylogeny reported here (**Figure 1**) strongly suggests that the *iaaL* gene has been horizontally exchanged among *P. savastanoi* strains. Since *iaaL* was shown to be carried by plasmids in certain *P. savastanoi* strains (Comai and Kosuge, 1980; Caponero et al., 1995; Pérez-Martínez et al., 2007), and because diverse virulence plasmids of *P. savastanoi* pv. savastanoi were shown to be readily transferred by conjugation to other *P. savastanoi* and *P. syringae* strains (Añorga et al., 2022), it is likely that this horizontal transfer of *iaaL* has been mediated by plasmids.

### 4.5 Role of the *iaaL* gene in the virulence of *P. syringae* complex strains

Previous studies have reported on the role of *iaaL* in the virulence of Pto DC3000 and Psn strains. However, the impact of this gene in virulence is strain-dependent, even among strains of the same pathovar, as *iaaL* mutants can be both hypovirulent and hypervirulent (Glass and Kosuge, 1988; Castillo-Lizardo et al., 2015; Cerboneschi et al., 2016). With this in mind, we tested whether the expression of the active allozymes IaaL_Psn-1_ and IaaL_Psf-3_ influenced the virulence of Psv NCPPB 3335 in olive plants or its ability to infect oleander plants. However, significant changes in the virulence and host range were not observed (data not shown). Psv NCPPB 3335 encodes a large suite of virulence and host-specificity factors that act collectively (Caballo-Ponce et al., 2017; Moreno-Pérez et al., 2020), it is therefore not surprising that the modification of just one of these factors in a strain not producing IAA–Lys induces significant changes in these phenotypes.

## 5 Conclusions

In conclusion, our analysis of allelic variation in the auxin-related *iaaL* gene of *P. savastanoi* identified a novel allele (*iaaL*_Psf_) exclusive of ash strains and showed that strains isolated from the same host share the same array of *iaaL* alleles, including Psr strains, which encode a single *iaaL*_Psn_ allele. We also show that olive and ash *P. savastanoi* strains can produce IAA–Lys, and that its production depends on both the concentration of pathogen-produced IAA and the codification of the alloenzymes IaaL_Psn-1_ or IaaL_Psf_. Our biochemical analyses confirm the functionality and specificity of lysine as a substrate of several IaaL allozymes. Additionally, an ash-specific IaaL variant exhibited a higher catalytic efficiency for both substrates (IAA and lysine) than its ortholog (IaaL_Psn-1_) encoded in tumorigenic strains. Finally, we show that the IAA–Lys synthase activity of IaaL_Psn-1_ was abolished by the insertion of two additional tyrosine residues, mirroring the corresponding insertion in the inactive allozyme IaaL_Psv-1_. Our results highlight the relevance of the allelic variation in the modulation of the functionality of a virulence gene related with the production of a bacterial phytohormone.

## Data availability statement

The original contributions presented in the study are included in the article/**Supplementary Material**. Further inquiries can be directed to the corresponding author.

## Author contributions

AP, VP, SL, VF, and CR planned and designed the research and analyzed the data. AP, HD-C, VP, and MV performed the experiments. AP, HD-C, SL, and CR designed and prepared the figures and tables. AP and CR wrote the manuscript with the help of SL. HD-C, VP, VF, and SL contributed to the revision of the manuscript. All authors contributed to the article and approved the submitted version.
