# Supplementary material for: Allelic variation in the indoleacetic acid-lysine synthase gene of the bacterial pathogen Pseudomonas savastanoi and its role in auxin production
Source: Front Plant Sci. 2023 Jun 6;14:1176705. doi: 10.3389/fpls.2023.1176705 (PMC10280071; doi:10.3389/fpls.2023.1176705)
Supplement: Supplementary file 1 [file DataSheet_1.pdf]

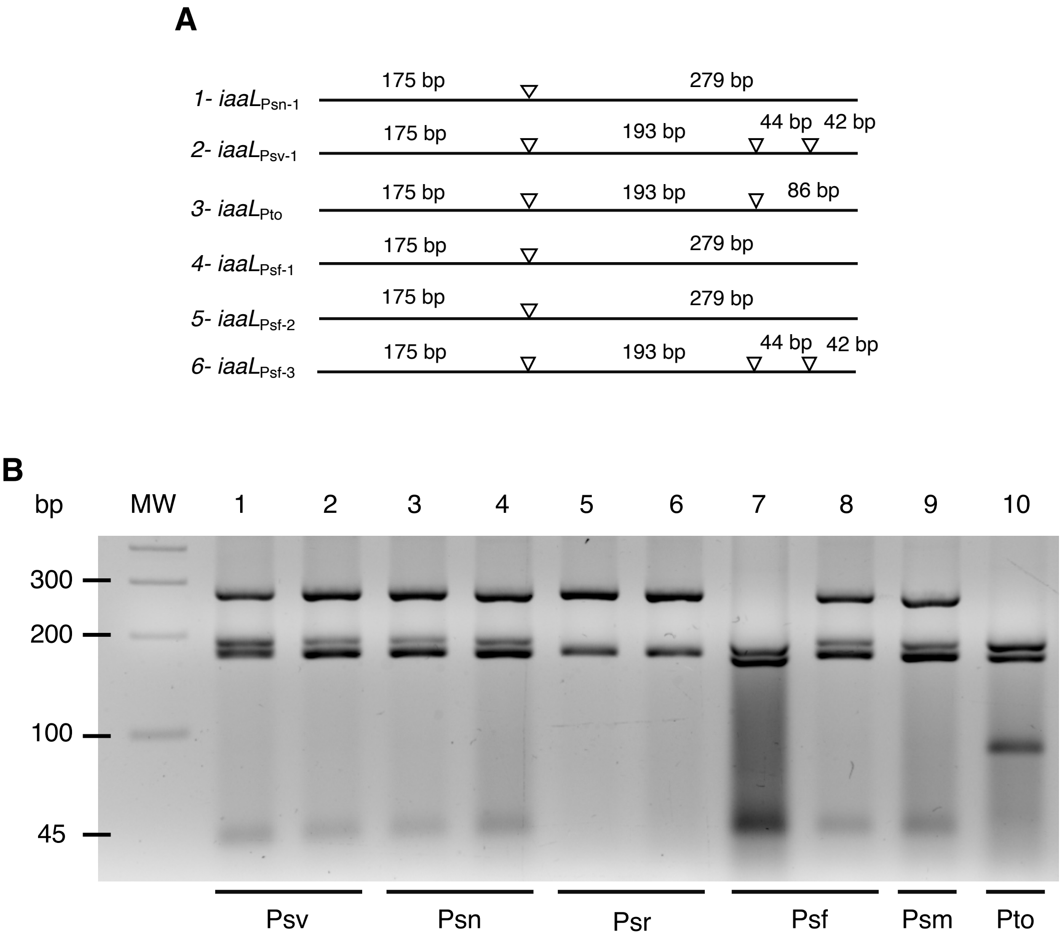

Figure S1. PCR-RFLP of *iaaL* genes encoded in *P. savastanoi* pathovars of woody hosts and *P. syringae* pv. tomato (Pto). **(A)** Schematic representation of an internal fragment of *iaaL* alleles amplified by PCR using primers iaaL-F-221 and iaaL-R-696 (Matas et al., 2009). HaeIII are indicated by arrowheads. **(B)** Gel electrophoresis (3% agarose) of HaeIII-digested amplicons. Lane 1, Psv NCPPB 3335; lane 2, Psv DAPPG 772; lane 3, Psn *Psn23*; lane 4, Psn CFBP 5067; lane 5, Psr CECT 4861; lane 6, Psr ICMP 16947; lane 7, Psf NCPPB 1006; lane 8, Psf CFBP 5062; lane 9, Psm Ph3; lane 10, Pto DC3000. MW, molecular size marker.

```
                                                                                                          70
IaaLPsn  IaaLPsn-1 MTAYDMEKEW SRISSTAAKI HQNNDFEGFT YQDFRTHVPI MDKDGFAAQT ERCLERNERN CLIGFTSGTS
         IaaLPsn-2 ---------Y SRISSTAAKI HQNNDFEGFT YQDFRTHVPI MDKDGFAAQT ERCLERNERN CLIGFTSGTS
         IaaLPsf-1 MTAYDMEKEW SRISSTAAKI HQNNDFEGFT YQDFRTHVPI MDKDGFAAQT ERCLERNERN CLIGFTSGTS
IaaLPsf  IaaLPsf-2 MTAYDMEKEW SRISSTAAKI HQNNDFEGFT YQDFRTHVPI MDKDGFAAQT ERCLERNERN CLIGFTSGTS
         IaaLPsf-3 MTAYDMEKEW SRISSTAAKI HQNNDFEGFT YQDFRTHVPI MDKDGFAAQT ERCLERNERN CLIGFTSGTS
         IaaLPsv-1 MTAYDVEKEW SRISNTAAKT HQNNDFEGFT YQNFRKHVPI MDKEGFTAQT ERCLERNERN CLIGFTSGTS
         IaaLPsv-2 MTAYDVEKEW SRISNTAAKT HQNNDFEGFT YQNFRKHVPI MDKEGFTAQT ERCLERNERN CLIGFTSGTS
         IaaLPsv-3 MTAYDVEKEW SRISNTAAKT HQNNDFEGFT YQNFRKHVPI MDKEGFTAQT ERCLERNERN CLIGFTSGTS
IaaLPsv  IaaLPsv-4 ------EKEW SRISNTAAKT HQNNDFEGFT YQNFRKHVPI MDKEGFTAQT ERCLERNERN CLIGFTSGTS
         IaaLPsv-5 MTAYDVEKEW SRISNTAAKT HQNNDFEGFT YQNFRKHVPI MDKEGFTAQT ERCLERNERN CLIGFTSGTS
         IaaLPsv-6 MTAYDVEKEW SRISNTAAKT HQNNDFEGFT YQNFRKHVPI MDKEGFTAQT ERCLERNERN CLIGFTSGTS
         IaaLPto   MTAYDVEKEW SRISNTAAKT HQNNDFEGFT YQDFRTHVPI MDKEGFAAQT ERCLERNERN CLIGFTSGTS
                                                                                                         140
IaaLPsn  IaaLPsn-1 GNIKRCYYYY --DCEVDE-- -DSSLSNVFR SNGFILPGDR CANLFTINLF SALNNTITMM AGNCGAHVVS
         IaaLPsn-2 GNIKRCYYYY --DCEVDE-- -DSSLSNVFR SNGFILPGDR CANLFTINLF SALNNTITMM AGNCGAHVVS
         IaaLPsf-1 GNLKRCYYYY --DCEVDK-- -DSSLSNVFR SNGFILPGDR CANLFTINLF SALNNMITMM AGNCGAHVVS
IaaLPsf  IaaLPsf-2 GNLKRCYYYY --DCEVDK-- -DSSLSNVFR SNGFILPGDR CANLFTINLF SALNNMITMM AGNCGAHVVS
         IaaLPsf-3 GNLKRCYYYY --DCEVDK-- -DSSLSNVFR SNGFILPGDR CANLFTINLF SALNNMITMM AGNCGAHVVS
         IaaLPsv-1 GNLKRCYYYY YYDCEVDE-- -DSSRSNVFR SNGFIQPGDR CANLFTINLF SALNNITTMM AGNCGAHVVS
         IaaLPsv-2 GNLKRCYYYY --DCEVDE-- -DSSRSNVFR SNGFIQPGDR CANLFTINLF SALNNITTMM AGNCGAHVVS
         IaaLPsv-3 GNLKRCYYYY --DCEVDE-- -DSSRSNVFR SNGFIQPGDR CANLFTINLF SALNNITTMM AGNCGAHVVS
IaaLPsv  IaaLPsv-4 GNLKRCYYYY --DCEVDEVD EDSSRSNVFR SNGFIQPGDR CANLFTINLF SALNNITTMM AGNCGAHVVS
         IaaLPsv-5 GNLKRCYYYY --DCEVDEVD EDSSRSNVFR SNGFIQPGDR CANLFTINLF SALNNITTMM AGNCGAHVVS
         IaaLPsv-6 GNLKRCYYYY --DCEVDE-- -DSSLSNVFR SNGFIQPGDR CANLFTINLF SALNNITTMM AGNCGAHVVS
         IaaLPto   GNLKRCYYYY --DCEVDE-- -DSSRSNVFR SNGFIQPGDR CANLFTINLF SALNNITTMM AGNCGAHVVS
                                                                                                         210
IaaLPsn  IaaLPsn-1 VGDITLVTKS HFEALNSIKL NVLLGVPSTI LQFINAMQHN GVHINIEKVV FTGESLKTFQ KKIIRQAFGE
         IaaLPsn-2 VGDITLVTKS HFEALNSIKL NVLLGVPSTI LQFINAMQHN GVHINIEKVV FTGESLKTFQ KKIIRQAFGE
         IaaLPsf-1 VGDITLVTKS HFEALNSIKL NVLLGVPSTI LQFINAMQHN GVHINIEKVV FTGESLKTFQ KKIIRQAFGE
IaaLPsf  IaaLPsf-2 VGDITLVTKS HFEALNSIKL NVLLGVPSTI LQFINAMQHN GVHINIEKVV FTGESLKTFQ KKIIRQAFGE
         IaaLPsf-3 VGDITLVTKS HFEALNSIKL NVLLGVPSTI LQFI AMQQH GVHIEIEKVV FNGEGLKTFQ KKIIREAFGQ
         IaaLPsv-1 VGDITLLTKS HFEALNSIKL NVLLGVPSTI LQFIDAMQQH GVHIEIEKVV FNGEGLKTFQ KKIIREAFGQ
         IaaLPsv-2 VGDITLLTKS HFEALNSIKL NVLLGVPSTI LQFIDAMQQH GVHIEIEKVV FNGEGLKTFQ KKIIREAFGQ
         IaaLPsv-3 VGDITLLTKS HFEALNSIKL NVLLGVPSTI LQFIDAMQQH GVHIEIEKVV FNGEGLKTFQ KKIIREAFGQ
IaaLPsv  IaaLPsv-4 VGDITLLTKS HFEALNSIKL NVLLGVPSTI LQFIDAMQQH GVHIEIEKVV FNGEGLKTFQ KKIIREAFGQ
         IaaLPsv-5 VGDITLLTKS HFEALNSIKL NVLLGVPSTI LQFIDAMQQH GVHIEIEKVV FNGEGLKTFQ KKIIREAFGQ
         IaaLPsv-6 VGDITLLTKS HFEALNSIKL NVLLGVPSTI LQFIDAMQQH GVHIEIEKVV FNGEGLKTFQ KKIIREAFGQ
         IaaLPto   VGDITLLTKS HFEALNSIKL NVLLGVPSTI LQFIDAMQQH GVHIDIEKVV FNGEGLKTFQ KKIIREAFGE
                                                                                                         280
IaaLPsn  IaaLPsn-1 QVSIVGVYGS SEGGILGFTN SPCHTEYEFL SDKYFIEKEG DSILITSLTR ENFTPLLRYR LGDTATLSMK
         IaaLPsn-2 QVSIVGVYGS SEGGILGFTN SPCHTEYEFL SDKYFIEKEG DSILITSLTR ENFTPLLRYR LGDTATLSMK
         IaaLPsf-1 QVSIVGVYGS SEGGILGFTN SPCHTEYEFL SDKYFIEKEG DSILITSLTR ENFTPLLRYR LGDTATLSM 
IaaLPsf  IaaLPsf-2 QVSIVGVYGS SEGGILGFTN SPCHTEYEFL SDKYFIEKEG DSILITSLTR ENFTPLLRYR LGDTATLSMK
         IaaLPsf-3 QVSIVGVYGS SEGGILGFTN SPCHTEYEFL SDKYFIEKEG DSILITSLTR ENFTPLLRYR LGDTATLSMK
         IaaLPsv-1 QVSIVGVYGS SEGGILGFTN SPCHTEYEFL SDKYFIEKEG DSILITSLTR ENFTPLLRYR LGDTATLSMK
         IaaLPsv-2 QVSIVGVYGS SEGGILGFTN SPCHTEYEFL SDKYFIEKEG DSILITSLTR ENFTPLLRYR LGDTATLSMK
         IaaLPsv-3 QVSIVGVYGS SEGGILGFTN SPCHTEYEFL SDKYFIEKEG DSILITSLTR ENFTPLLRYR LGDTATLSMK
IaaLPsv  IaaLPsv-4 QVSIVGVYGS SEGGILGFTN SPCHTEYEFL SDKYFIEKEG DSILITSLTR ENFTPLLRYR LGDTATLSMK
         IaaLPsv-5 QVSIVGVYGS SEGGILGFTN SPCHTEYEFL SDKYFIEKEG DSILITSLTR ENFTPLLRYR LGDTATLSMK
         IaaLPsv-6 QVSIVGVYGS SEGGILGFTN SPCHTEYEFL SDKYFIEKEG DSILITSLTR ENFTPLLRYR LGDTATLSMK
         IaaLPto   QVSIVGVYGS SEGGILGFTN SPCHTEYEFL SDKYFIEKEG DSILITSLTR ENFTPLLRYR LGDTATLSLK
                                                                                                         350
IaaLPsn  IaaLPsn-1 GDKLYLTDIQ REDMSFNFMG NLIGLGIIQQ TIKQTLGRSL EIQVHLSVTE ERKELVTVFV QASEVDEDER
         IaaLPsn-2 GDKLYLTDIQ REDMSFNFMG NLIGLGIIQQ TIKQTLGRSL EIQVHLSVTE ERKELVTVFV QASEVDEDER
         IaaLPsf-1 GDKLYLTDIQ REDMSFNFMG NLIGVGIIQH AIKQTLGRSL EIQVHLSVTE ERKELVTVFV QASEVDEDER
IaaLPsf  IaaLPsf-2 GDKLYLTDIQ REDMSFNFMG NLIGLGIIQQ AIKQTLGRPL EIQVHLSVTE ARKELVTVFV QASEVDEEER
         IaaLPsf-3 GDKLYLTDIQ REDMSFNFMG NLIGLGIIQQ AIKQTLGRPL EIQVHLSVTE ARKELVTVFV QASEVDEEER
         IaaLPsv-1 GDKLYLTDIQ REDMSFNFMG NLIGLGIIQQ AIKQTLGRPL EIQVHLSVTE ARKELVTVFV QASEVDEEER
         IaaLPsv-2 GDKLYLTDIQ REDMSFNFMG NLIGLGIIQQ AIKQTLGRPL EIQVHLSVTE ARKELVTVFV QASEVDEEER
         IaaLPsv-3 GDKLYLTDIQ REDMSFNFMG NLIGLGIIQQ AIKQTLGRPL EIQVHLSVTE ARKELVTVFV QASEVDEEER
IaaLPsv  IaaLPsv-4 GDKLYLTDIQ REDMSFNFMG NLIGLGIIQQ AIKQTLGRPL EIQVHLSVTE ARKELVTVFV QASEVDEEER
         IaaLPsv-5 GDKLYLTDIQ REDMSFNFMG NLIGLGIIQQ AIKQTLGRPL EIQVHLSVTE ARKELVTVFV QASEVDEEER
         IaaLPsv-6 GDKLYLTDIQ REDMSFNFMG NLIGLGIIQQ AIKQTLGRPL EIQVHLSVTE ARKELVTVFV QASEVDEEER
         IaaLPto   GDKLYLTDIQ REDMSFNFMG NLIGLGIIQQ AIKQTLGRTL EIQVHLSVTD ARKELVTVFV QASEVNEDER
                                                                     400
IaaLPsn  IaaLPsn-1 VRIETAIADI PDIKEAYQKN QGSVSVLRKD ARDYAVSERG KMLYIIDRRN
         IaaLPsn-2 VRIETAIADI PDIKEAYQKN QGSVSVLRKD ARDYAVSERG KMLYIIDRRN
         IaaLPsf-1 ARIETAIADI PDINEAYQKD QGSVSVLRKD ARDYAVSERG KMLYIIDRRN
IaaLPsf  IaaLPsf-2 ARIETAIADI PDINEAYQKD QGSVSVLRKD ARDYAVSERG KMLYIIDRRN
         IaaLPsf-3 ARIETAIADI PDINEAYQKD QGSVSVLRKD ARDYAVSERG KMLYIIDRRN
         IaaLPsv-1 ARIETAIADI PDINEAYQKD QGSVSVLRKD ARDYAVSERG KMLYIIDRRN
         IaaLPsv-2 ARIETAIADI PDINEAYQKD QGSVSVLRKD ARDYAVSERG KMLYIIDRRN
         IaaLPsv-3 ARIETAIADI PDINEAYQKD QGSVSVLRKD ARDYAVSERG KMLYIIDRRN
IaaLPsv  IaaLPsv-4 ARIETAIADI PDINEAYQKD QGSVSVLRKD ARDYAVSERG KMLYIIDRRN
         IaaLPsv-5 ARIETAIADI PDINEAYQKD QGSVLVLRKD ARDYAVSERG KMLYIIDRRN
         IaaLPsv-6 ARIETAIADI PDINEAYQKD QGSVSVLRKD ARDYAVSERG KMLYIIDRRN
         IaaLPto   ARIETAIADI PDINEAYQKD QGSVLVVRKD ARDYAVSERG KMLYIIDRRN
```

Figure S2. Multiple alignment of IaaL amino acid sequences encoded by *iaaL*$_{\text{Psf}}$, *iaaL*$_{\text{Psv}}$, *iaaL*$_{\text{Psn}}$ and *iaaL*$_{\text{Pto}}$ alleles in *P. savastanoi* strains. The alignment of the amino acid sequences was performed with MEGAX (Kumar et al., 2018) using the ClustalW algorithm. Exclusive amino acid residues encoded in all allozymes of the same group are shown in blue (IaaL$_{\text{Psv}}$), red (IaaL$_{\text{Psn}}$), yellow (IaaL$_{\text{Psf}}$) and green (IaaL$_{\text{Pto}}$); gray-colored boxes indicate residues varying within allozymes of the same group.

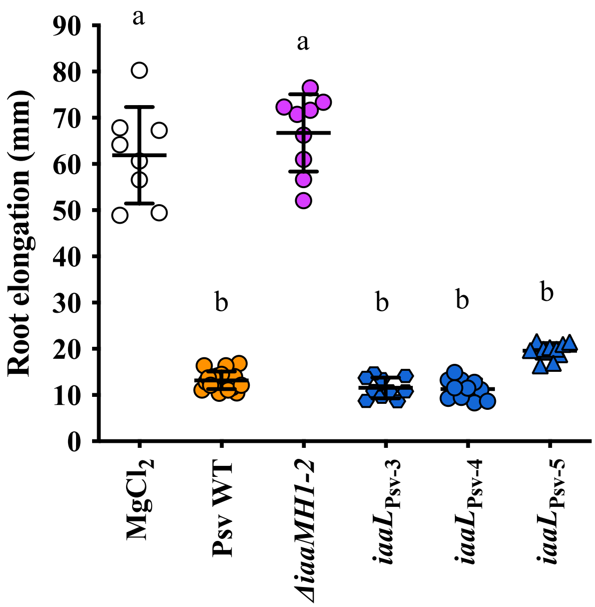

Figure S3. Root elongation in *A. thaliana* Col-0 seedlings grown on vertical plates in the presence of Psv NCPPB 3335 strains overexpressing diverse *iaaL* alleles from the constitutive promoter *nptII.* The strains Psv NCPPB 3335 (WT) and Psv *ΔiaaMH1-2* (*ΔiaaMH1-2*) were used as control for high and low production of free IAA, respectively. The bars represent the mean ± standard deviation for 9 to 15 seedlings.

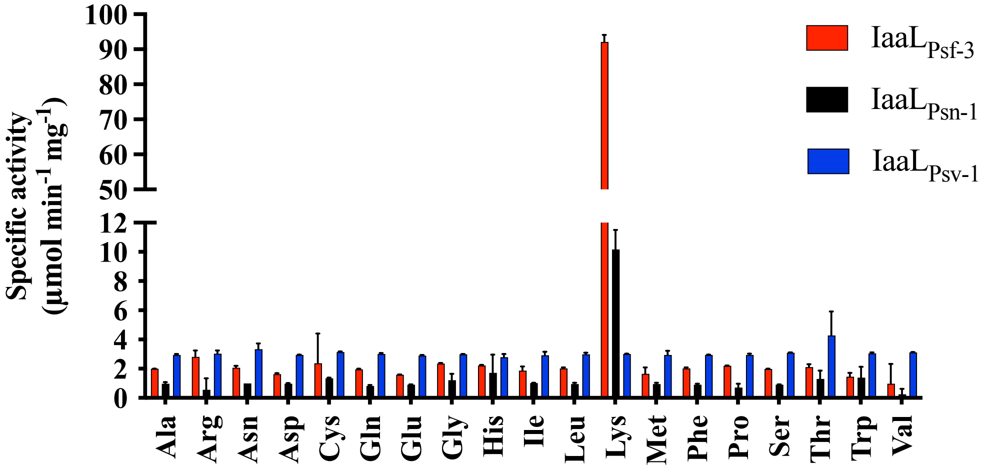

Figure S4. Specific activity of IaaL proteins encoded in the *iaaL*$_{Psf\text{-}3}$, *iaaL*$_{Psn\text{-}1}$ and *iaaL*$_{Psv\text{-}1}$ alleles from Psf NCPPB 1006, Psn *Psn23* and Psv NCPPB 3335, respectively. The biochemical assay was performed in the presence of 1mM IAA and 5mM of the amino acid. Bars represent the mean ± standard deviation of two independent experiments.
