# Supplementary material for: Allelic variation in the indoleacetic acid-lysine synthase gene of the bacterial pathogen Pseudomonas savastanoi and its role in auxin production
Source: Front Plant Sci. 2023 Jun 6;14:1176705. doi: 10.3389/fpls.2023.1176705 (PMC10280071; doi:10.3389/fpls.2023.1176705)
Supplement: Supplementary file 2 [file DataSheet_2.pdf]

Supplementary Material

Allelic variation in the indole acetic acid-lysine synthase gene in the bacterial pathogen *Pseudomonas savastanoi* reflects adaptations to the plant host and the concentration of pathogen-produced auxin

Adrián Pintado, Hilario Domínguez-Cerván, Victoria Pastor, Marissa Vincent, Soon Goo Lee, Víctor Flors, Cayo Ramos

*** Correspondence:** Cayo Ramos, crr@uma.es

Table S1. Bacterial strains used in this work.

Strains[a]	Relevant characteristics (host)[b]	References
Pseudomonas savastanoi & Pseudomonas syringae		
Psv NCPPB 3335	Wild-type strain (*Olea europaea*)	(Pérez-Martínez et al., 2007)
Psv *ΔiaaMH1-2*	Double deletion mutant *iaaMH1* e *iaaMH2*	(Aragón et al., 2014)
Psv DAPP-PG772	Wild-type strain (*O. europaea*)	(Hosni et al., 2011)
Psn *Psn23*	Wild-type strain (*Nerium oleander*)	(Tegli et al., 2011)
Psn CFBP 5067	Wild-type strain (*N. oleander*)	(Janse, 1991)
Psf NCPPB 1006	Wild-type strain (*Fraxinus excelsior*)	(Janse, 1981)
Psf CFBP 5062	Wild-type strain (*F. excelsior*)	(Janse, 1991)
Pto DC3000	Wild-type strain (*Solanum lycopersicum*)	(Cuppels, 1986)
Escherichia coli		
DH5α	F^-, ϕ80d*lacZΔ*M15, *Δ*(*lacZYA-argF*)U169, *deoR*, *recA*1 *endA*1, *hsdR*17(r_K^- m_K^+), *phoA*, *supE*44, λ^-, *thi*-1, gyrA96, *rel*A1	(Hanahan, 1983)
BL21	F^- *ompT hsdS*$_B$(r_B^- m_B^-) *gal dcm* (DE3)	(Studier and Moffatt, 1986)

[a]Psv, Psn and Psf: *P. savastanoi* pv. savastanoi, pv. nerii and pv. fraxini, respectively. Pto: *P. syringae* pv. syringae.

[b]km^R, kanamycin resistance; *Olea europaea*, olive; *Nerium oleander*, oleander; *Fraxinus excelsior*, ash; *Solanum lycopersicum*; tomato.

Table S2. Plasmids used in this work.

Name	Relevant characteristics	References
pAMEX	Broad-host-range expression vector with *nptII* promoter (Km^R)	Macho et al., 2009
pAMEX::*iaaL*$_{Psn-1}$	pAMEX derivative, contains the *iaaL*$_{Psn-1}$ allele (Km^R)	This work
pAMEX::*iaaL*$_{Psf-1}$	pAMEX derivative, contains the *iaaL*$_{Psf-1}$ allele (Km^R)	This work
pAMEX::*iaaL*$_{Psf-3}$	pAMEX derivative, contains the *iaaL*$_{Psf-3}$ allele (Km^R)	This work
pAMEX::*iaaL*$_{Psv-1}$	pAMEX derivative, contains the *iaaL*$_{Psv-1}$ allele (Km^R)	This work
pAMEX::*iaaL*$_{Psv-2}$	pAMEX derivative, contains the *iaaL*$_{Psv-2}$ allele (Km^R)	This work
pAMEX::*iaaL*$_{Psv-3}$	pAMEX derivative, contains the *iaaL*$_{Psv-3}$ allele (Km^R)	This work
pAMEX::*iaaL*$_{Psv-4}$	pAMEX derivative, contains the *iaaL*$_{Psv-4}$ allele (Km^R)	This work
pAMEX::*iaaL*$_{Psv-5}$	pAMEX derivative, contains the *iaaL*$_{Psv-5}$ allele (Km^R)	This work
pAMEX::*iaaL*$_{Pto}$	pAMEX derivative, contains the *iaaL*$_{Pto}$ allele (Km^R)	This work
pET28a	His-tag protein expression in *E. coli* (Km^R)	Novagen, USA
pET28a::*iaaL*$_{Psn-1}$	pET28a derivative, contains the *iaaL*$_{Psn-1}$ allele (Km^R)	This work
pET28a::*iaaL*$_{Psf-1}$	pET28a derivative, contains the *iaaL*$_{Psf-1}$ allele (Km^R)	This work
pET28a::*iaaL*$_{Psf-3}$	pET28a derivative, contains the *iaaL*$_{Psf-3}$ allele (Km^R)	This work
pET28a::*iaaL*$_{Pto}$	pET28a derivative, contains the *iaaL*$_{Pto}$ allele (Km^R)	This work
pET28a::*iaaL*$_{Psv-1}$	pET28a derivative, contains the *iaaL*$_{Psv-1}$ allele (Km^R)	This work
pET28a::*iaaL*$_{Psv-2}$	pET28a derivative, contains the *iaaL*$_{Psv-2}$ allele (Km^R)	This work
pET28a::*iaaL*$_{Psv-3}$	pET28a derivative, contains the *iaaL*$_{Psv-3}$ allele (Km^R)	This work
pET28a:: *iaaL*$_{PsnYY}$	pET28a derivative, contains the *iaaL*$_{Psn-1}$ allele modified by site-directed mutagenesis (Y_{81}, Y_{82}) (Km^R)	This work

Table S3. Primers used in this work.

Name	Sequence (5' – 3')[a]	Restriction enzyme
**Construction of pAMEX-derivative plasmids for heterologous expression**		
iaaL-RBS-F	TTAAATAAGCTTAATCCACGTTTTGCCCACC	HindIII
iaaL-RBS-R	TTAAATGAATTCTCAGTTTCGGCGGTCGATG	EcoRI
**Construction of pET28a-derivative plasmids for protein expression**		
*iaaL*Psn23PC-F	TTAAATGGATCCAATGACTGCCTACGATATGG	BamHI
*iaaL*Psn23PC-R	TTAAATGCGGCCGCGTTTCGGCGGTCGATG	NotI
*iaaL*Psv48PC-F	TTAAATGGATCCAATGACTGCCTACGATGTAG	BamHI
*iaaL*PtoPC-F	TTAAATGGATCCAATGACTGCCTACGATGTA	BamHI
*iaaL*PtoPC-R	TTAAATGCGGCCGCTCAATTCCTGCGGTCA	NotI
pET-UP	AGATCTCGATCCCGC	-
pET-DOWN	TTTGTTAGCAGCCGG	-
**Site-directed mutagenesis of *iaaL*$_{Psn-1}$**		
Mut_YY_F	CGCTGTTATTACTACTACTACTACGACTGCGA<br>A<br>GTCGATGA	-
Mut_YY_R	TCATCGACTTCGCAGTCGTAGTAGTAGTAGTA<br>A<br>TAACAGCG	-
**PCR-RFLP**		
iaaL-F-221	GGCACCAGCGGCAACATCAA	-
iaaL-R-696	CGCCCTCGGAACTGCCATAC	-
**RT-qPCR**		
*iaaL*2 F-175	ACGCTGTCTGGAACGCAACGAGC	-
*iaaL*2 R-271	GCTGCGGAAGACGTTGGAGCGTG	-
qRT-*iaaL*Psn-F	AACAAGTCTCCATCGTCGGT	-
qRT-*iaaL*Psn-R	CGTATTCAGTGTGGCAAGGG	-
qRT-*iaaL*Psf-F	TCTGCTCTGAACAACATG	-
qRT-*iaaL*Psf-R	AGTACGTTGAGCTTGATC	-
gyrA-F	GACGAGCTGAAGCAGTCCTACC	-
gyrA-R	TTCCAGTCGTTACCCAGCTCG	-

[a]Underlined nucleotides show the sequences recognized by the indicated restriction enzyme.
